# Successful Implementation Strategies in iCARE Nigeria—A Pilot Intervention with Text Message Reminders and Peer Navigation for Youth Living with HIV

**DOI:** 10.3390/tropicalmed8110498

**Published:** 2023-11-16

**Authors:** Aima A. Ahonkhai, Kehinde M. Kuti, Lisa R. Hirschhorn, Lisa M. Kuhns, Robert Garofalo, Amy K. Johnson, Adedotun Adetunji, Baiba Berzins, Ogochukwu Okonkwor, Olutosin Awolude, Olayinka Omigbodun, Babafemi O. Taiwo

**Affiliations:** 1 Department of Medicine, Infectious Diseases, Vanderbilt University Medical Center, Nashville, TN 37203, USA; 2 Vanderbilt Institute for Global Health, Vanderbilt University Medical Center, Nashville, TN 37203, USA; 3 Department of Medicine, Infectious Diseases, Medical Practice Evaluation Center, Massachusetts General Hospital, Boston, MA 02114, USA; 4 Department of Family Medicine, University College Hospital, Ibadan 200005, Nigeria; bolanle22@yahoo.com (K.M.K.);; 5 Department of Medical Social Sciences, Feinberg School of Medicine, Northwestern University, Chicago, IL 60611, USA; lisa.hirschhorn@northwestern.edu; 6 Department of Pediatrics, Northwestern University Feinberg School of Medicine, Chicago, IL 60611, USA; lkuhns@luriechildrens.org (L.M.K.); rgarofalo@luriechildrens.org (R.G.); akjohnson@luriechildrens.org (A.K.J.); 7 Division of Adolescent and Young Adult Medicine, Ann & Robert H. Lurie Children’s Hospital of Chicago, Chicago, IL 60611, USA; 8 Department of Preventive Medicine, Northwestern University Feinberg School of Medicine, Chicago, IL 60611, USA; 9 Division of Infectious Diseases, Northwestern University, Chicago, IL 60611, USAogochukwu@northwestern.edu (O.O.);; 10 Department of Obstetrics and Gynecology, College of Medicine, University of Ibadan, Ibadan 200212, Nigeria; tosinawolude@yahoo.com; 11 Infectious Disease Institute, College of Medicine, University of Ibadan, Ibadan 200212, Nigeria; 12 Department of Psychiatry, College of Medicine, University of Ibadan, Ibadan 200212, Nigeria; olayinka.omigbodun@gmail.com

**Keywords:** HIV, Nigeria, implementation science, peer navigation, retention in care, adolescents and young adults

## Abstract

To address poor outcomes among adolescents and young adults living with HIV (AYA-HIV), iCARE Nigeria successfully piloted two-way text message antiretroviral therapy (ART) reminders together with peer navigation. Study participants had significant improvement in ART adherence and viral suppression at 48 weeks. Understanding facto of this intervention. We used explanatory, mixed methods to assess implementation outcomes (feasibility, acceptability, and adoption) and identify implementation strategies used or adapted to promote intervention success. Quantitative data included participant surveys, program records, and back-end mHealth data, and were summarized using descriptive statistics. Qualitative data were collected from key informants and focus group discussions with program staff and summarized using directed content analysis. iCARE Nigeria was feasible as evidenced by ease of recruitment, high retention of patients and peer navigators (PN), and successful deployment of initial text message reminders (99.9%). Most participants (95%) and PN (90%) found text message reminders were not bothersome or intrusive. Implementation strategies employed to facilitate intervention success included: (1) selecting, training, supervising, and matching of PN to patients; (2) tailoring frequency (daily to weekly) and mode of communication between PN and patients according to patient need; (3) routine screening for adherence challenges; (4) changing phone airtime stipends from monthly to weekly in response to rapid depletion; and (5) conducting telecommunication needs assessments, to identify and troubleshoot implementation barriers (issues with mobile devices, power availability). iCARE Nigeria was feasible and acceptable with high adoption by stakeholders. The implementation strategies identified here can be tailored for intervention scale-up in similar environments to promote ART adherence for AYA-HIV.

## 1. Introduction

Adolescents and young adults have been increasingly recognized as a key population for intervention among people with HIV (PWH) [1]. Adolescents and young adults living with HIV (AYA-HIV) have not seen the same benefits from the scale-up of highly effective antiretroviral therapy (ART) as in either older adults or younger children [2,3,4]. Due to complex developmental, psychosocial, and physiologic factors (some representing sequalae of HIV infection), AYA-HIV tends to show poor engagement in HIV care and adherence to ART leading subsequently to poor virologic control [5]. Nigeria, the most populous African country, is home to nearly one-tenth of all youth living with HIV worldwide. Only 33% of AYA-HIV in the country have achieved virologic suppression [3,6,7]. Nigeria is therefore an ideal environment to assess novel interventions to improve viral suppression among AYA-HIV [8].

The Intensive Combination Approach to Roll Back the Epidemic in Nigerian Adolescents Study (iCARE Nigeria) is a multi-faceted HIV treatment intervention integrating two evidence-based approaches: mobile health (mHealth) technology and peer navigation [9]. Incorporating a locally adapted version of a CDC-recommended mHealth adherence intervention TXTXT, and WHO-recommended peer navigation, the treatment intervention consisted of daily, personalized, bi-directional text message reminders with peer navigation and support for AYA-HIV in Nigeria [9]. In the single-arm pilot trial, participants (n = 40) showed significant improvement in viral suppression from baseline (35% virally suppressed) to 24 weeks (68% virally suppressed) and 48 weeks (60% virally suppressed) [10]. In the ongoing second phase of the study, the intervention has been scaled up to five additional sites in Nigeria for evaluation in a cluster randomized trial design [11].

Implementation science provides helpful frameworks to guide the identification of important factors to consider and strategies to use when implementing evidence-based interventions across different community contexts. Implementation strategies are defined as the “*methods or techniques used to enhance the adoption, implementation, and sustainability of a clinical program or practice*” [12]. Since implementation strategies represent the specific recipe of methods that define the “how-to” of getting the intervention into practice, it is important to identify these strategies to guide replication and scale-up of successful interventions [12]. There have been increased efforts to name and specify implementation strategies for this purpose and to encourage researchers to document these strategies so that they can be replicated and adapted [12,13].

Reflecting the success of the iCARE Nigeria treatment intervention pilot, the objective of this study was to (1) further describe feasibility, acceptability, and adoption including the perspective of non-patient key stakeholders (namely peer navigators (PNs) and other program staff), and (2) identify and describe implementation strategies used or adapted to promote intervention success.

## 2. Materials and Methods

The study setting was the HIV clinic of the Infectious Disease Institute (IDI), College of Medicine, University of Ibadan, an established PEPFAR-supported HIV service provider since 2002. The IDI HIV clinic offers comprehensive HIV services including a dedicated adolescent and young adult HIV program for individuals aged 15–24 years.

We used a sequential, explanatory, mixed-methods study design to measure intervention and implementation outcomes for the iCARE Nigeria treatment arm, and qualitative methods to understand the implementation strategies contributing to these outcomes [14]. We used the Proctor framework to guide our assessment of implementation outcomes (feasibility, acceptability, adoption), from the program data and perspective of key program stakeholders including PNs, program managers, and study coordinators [12,13].

### 2.1. Quantitative Data Collection

Quantitative data included self-reported assessments, completed by study participants enrolled in the pilot trial between June 2019 and July 2020. Specifically, a telecommunication needs assessment and 10-item HIV stigma assessment validated for youth were completed at enrollment. Client Satisfaction Questionnaire-8 (for TXTXT and peer navigation components of the intervention) and survey questions (designed by the study team) assessing intervention acceptability were completed at the end of the 6-month pilot [15]. We additionally collected back-end data from the TXTXT intervention itself to determine the proportion of text reminders delivered and the proportion of messages to which participants responded. These were all summarized using descriptive statistics.

### 2.2. Qualitative Data Collection

Qualitative data were collected in person from key informant interviews (KIIs) and focus group discussions FDGs with program staff in November of 2021 (PNs, program manager, and study coordinators) using a semi-structured guide following the Proctor framework [12,13]. The study team comprised 1 program manager, 2 study coordinators, and 9 peer navigators in total (5 male, 4 female). KIIs were conducted in person by researchers (AA, LH) with expertise in implementation and qualitative research methods who were not involved in the iCARE pilot study, and who did not work in the HIV IDI clinic. Key informants were members of the study team who were chosen based on their roles in implementing the study. Patients were not included as key informants due to public health constraints posed by COVID-19. KIIs were conducted with PNs for ease of scheduling, and a FGD was conducted with study coordinators and program managers. PNs were purposively sampled, ensuring representation by gender with a plan to expand sampling if needed to reach thematic saturation (4 of 9 PNs were female). Interviews were conducted in a private office and audio recorded. Study participants provided informed consent prior to each interview which lasted 45–60 min.

### 2.3. Data Analysis

We used descriptive statistics for quantitative outcomes from the existing program data. For qualitative data, interviews and FGD were transcribed. We then applied explanatory mixed methods using the qualitative data [14]. Transcripts were coded by two team members and analyzed using directed content analysis to identify implementation strategies that facilitated the quantitative intervention success (quantitative outcomes) both with regard to effectiveness and implementation outcomes (feasibility, acceptability, adoption) using explanatory mixed methods. We also identified how the strategies were implemented and adapted to address implementation barriers. Implementation strategies were further described and categorized using established guidance [12]. Specifically, we described the strategy itself, the person enacting the strategy (actor), named the specific steps involved (action), named the action target, when the strategy was used (temporality), enumerated the dosage of the strategy, and described the implementation outcome affected [12]. Next, we assessed whether identified strategies aligned with the Expert Recommendations for Implementing Change (ERIC) taxonomy. The ERIC taxonomy was created by stakeholder experts to provide consensus and categorization of key implementation strategies [16].

## 3. Results

### 3.1. Quantitative Summary of Implementation Outcomes

iCARE Nigeria was feasible as evidenced by our ability to reach recruitment targets and sustain high retention of PNs. Eight PNs were initially recruited for the intervention, and an additional navigator was later trained as a backup. All were retained throughout the pilot intervention. Delivery of text message reminders was feasible in this low and middle-income (LMIC) environment. On average over the 12-month pilot study, 99.9% of daily text message reminders for their first or only daily ART dose were successfully sent to participants without error.

As published elsewhere, 40 participants completed the pilot trial and responded to the three survey assessments. The average age was 19.9 years, 50% (n = 20) identified as female, and 95% (n = 38) had secondary or tertiary education. The mode of HIV acquisition was perinatal in 55% (n = 22), non-perinatal in 32.5% (n = 13), and unknown in 12.5% (n = 5) [10]. The average baseline stigma score was 2.6, SD 0.5 (scale 1–5). Among the 19 participants who were on twice-daily ART regimens, 100% of their text message reminders for their second ART dose were sent without error. Furthermore, iCARE Nigeria was acceptable to participants. Most patients reported that the text message reminders (95%, n = 38/40) and PNs (90%, n = 36/40) were not bothersome or intrusive. Adoption of the SMS intervention was moderate. Participants replied on average to 42% of text message reminders (for the first/only daily ART dose) and 29% of the reminders for the second ART dose.

### 3.2. Qualitative Summary of Implementation Strategies Explaining Intervention and Implementation Success

One FGD was completed with one program manager and two study coordinators, and in-depth interviews were completed with three PNs (1 female and 2 male; 33% of PNs interviewed and 44% of PNs employed for the intervention identified as female). Additional interviews were not sought as thematic saturation was reached. Analysis revealed three categories of implementation strategies: core implementation strategies (defined a priori and codified in the intervention protocol) that either (1) contributed to favorable implementation and effectiveness outcomes, (2) directly helped address implementation barriers, or (3) were adapted or added to address barriers (Table 1).

### 3.3. Selection of Peer Navigators

PNs were recruited from engaged peer leaders in the HIV clinic where the intervention was based. This helped the study team identify highly motivated individuals who were already invested in providing peer support and had valuable experience in behaviors and skills associated with successful ART adherence. A peer navigator who was asked by one of the local study investigators whether he would be interested in joining the project as a peer navigator described:

“at some point she said something like, “you … have this experience and at a point you [can] give back to [your peers]”. I think that was the most touching part of it, like, I mean, I faced a lot of stuff… I was about 13 [or] thereabouts then when I started the medications, it was frustrating, it was challenging, I just wanted to be able to say, ok, let me see …as many youths I can also help”.

### 3.4. Training of Peer Navigators

PNs received an intensive two-day training prior to study commencement. The curriculum for this training was developed with input from youth and healthcare providers in the HIV clinic and included didactic sessions, group activities, telephone call role-play, and hands-on demonstration of the short message service (SMS) reminders. Topics included an introduction to HIV/AIDS, a discussion of common myths, ethical issues related to HIV care, privacy and confidentiality, professionalism, HIV prevention strategies, basic counseling and communication skills, telephone etiquette, mental health among AYA-HIV, sexual health and youth-friendly services. These topics and study protocols were comprehensively outlined in the study intervention manual which was affectionately called “the iCARE bible” by the study coordinators. Additional training occurred at week 24. One PN shared: “They train us, and they gave us booklet. So, if you … don’t understand anything you can go through that book and read again” [Female 2].

### 3.5. Matching Peer Navigators

In an effort to leverage shared experiences, PNs were matched with participants based on age, level of education, and religious background. Gender was not a primary consideration; PN did not identify gender differences as a barrier to their work. One PN who worked in a specialized professional field was matched with a participant who was training in a similar field. He described how she opened up in their relationship after appreciating their shared professional experiences.

### 3.6. Supervision of Peer Navigators

PN had ongoing supervision from several members of the team. They participated in weekly (virtual or face-to-face) meetings with the Study Coordinators, and bi-weekly meetings with the in-country lead clinician investigator. Topics for discussion included caseload, frequency of contact with participants, barriers to participant contact, troubleshooting of problems, job satisfaction, and needs for additional training or mentoring. This system allowed for ongoing assessment of PN needs, successes, prompt identification and addressing of challenges, and ensured fidelity to the study protocol.

One of the PNs shared: “So, most times with the [study coordinators] they are very good actually, they make the intervention very easy because they are very easy to meet and tell them about your peer challenges” [Male 1].

Further, these interactions helped PNs feel personally supported. One PN highlighted this by sharing that “So they tell us, “…it’s not only about iCARE; you too should come and share your experiences with us… you people can talk freely; you can interact with us freely. We are like your elder brother…Don’t see us as like workers…we are like your elder brothers you can talk to” [Male 2].

### 3.7. Flexibility in the Frequency/Mode of Communication

According to the protocol, PNs were instructed to communicate with their assigned participants at least once every two weeks via text or SMS message, social media, phone call, or face-to-face interaction. PNs found that they needed this flexible approach to contact participants and optimize communication. They described that some participants had very simple mobile phones that could only send or receive text messages or phone calls. Others had the ability to run WhatsApp, and could easily be pinged when they were online. One PN said: “… with social media you can easily just pop on WhatsApp, “ok, this person is online, I think I can call them now”.

While some modes of communication were more efficient, PNs also recognized that other modes of communication provided more pertinent information about the participant’s psychosocial circumstances. One PN shared:

“Yes…phone call is the best in my opinion, because you can be able to read what they might be saying, because like text messaging someone can tell you, “I’m fine”, meanwhile they might be shedding tears inside. That’s why I feel the phone is still my own best approach…”

[Male 2]

Early identification and intervention on adherence challenges by peer navigators.

Key elements of peer navigation during the intervention included regular communication to provide general health education, adherence counseling, referral to services (through the program managers), and crisis response. PNs recognized that regular communication with patients allowed them to identify adherence challenges, usually before they would have become evident to the clinical team, and often even before they impacted adherence. One PN described one of his participants:

“When we got in the first question she asked me… “how long have you been on the medication?” and I told her I’ve been on it 2005, then…That was storytelling. But the issue is, I told my story and she was like “wow!” And at some point we were always talking for hours per day. Then she…at that point I realized [because she told me] she wasn’t adherent, but she was just telling [other] people “yes, I am adherent”

[Male 2]

### 3.8. Core Implementation Strategies to Address Implementation Barriers

#### Needs Assessment

A needs assessment was conducted at baseline for all study participants to understand participant mobile phone use and practices, and gain insight into potential barriers to feasibility and successful adoption of the intervention. Participants were specifically queried on phone ownership, operating system, and functionality if regular electricity was available for charging the phone in the home, and, if not, where participants typically charged their devices. Findings were used to create an action plan to address communication barriers. For example, 23% (n = 9) of participants enrolled in the intervention had no mobile phones. Among those with phones, 26% (n = 8) described that their phones were faulty (e.g., broken or malfunctioning screen, weak battery). All study participants without phones and 7 participants with faulty phones were provided with basic phones for the duration of the intervention. The needs assessment also identified that 15% (n = 6) of participants did not have consistent electricity in their homes and described charging their phones at a neighbor’s house, at locations where a generator was available, or when they did not have electricity at home. As part of the strategy, these participants were provided with portable chargers and counseled on strategies to charge their phones. Without these assessments and subsequent action plans, the feasibility and fidelity of the SMS intervention would have been challenged.

Despite this assessment, inconsistent power availability remained a barrier to charging mobile phones for some. One PN shared: “Sometimes it’s challenging reaching out to them because…because they have to swap numbers and asides, because of the power issues in Nigeria, sometimes their phones go off for like a day or two and they’ll be like, ‘I did not charge my phone’” [Female 1].

### 3.9. Adaptation of Strategies to Address Implementation Barriers

#### Change in the Frequency of Provision of Monies for Airtime

Participants were provided with monthly phone airtime stipends (400 NGN, approximately USD 1) to facilitate receipt and response to SMS medication reminders. Though medication reminders were a major component of the iCARE Nigeria intervention, the study team quickly observed that participants would exhaust this airtime before the month ended. This practice meant participants could receive, but not respond to, their SMS reminders. To address this barrier, the study team dispensed the same total monthly amount of airtime distributed weekly instead of monthly. This approach ensured participants had some airtime each week. Indeed, PNs shared the trouble participants had holding onto airtime for a full month. One said: “Well…you know, sometimes… What I noticed about younger peers is that uh…because of, you know, airtime…because they tend to…when they have a phone, they tend to like get airtime on credit” [Female 1]. Airtime “purchased” on credit was immediately deducted from the funds paid later to recharge the phone’s airtime by the service provider.

## 4. Discussion

The iCARE Nigeria treatment intervention combined peer support with daily, customized, two-way SMS medication reminders to AYA-HIV in Nigeria. Our analysis showed this combination intervention was feasible in a LMIC setting and acceptable to program staff and PNs tasked with providing critical peer support. We found moderate adoption of the SMS intervention by AYA-HIV, though overall, the intervention was delivered with fidelity. Using explanatory mixed methods, we were able to identify a set of key bundles of strategies that helped overcome implementation barriers and were perceived to be responsible for implementation success. A clear understanding of these strategies is often missing from the literature but is a fundamental step for replication and scale-up.

We identified eight implementation strategies that contributed to both the implementation success and effectiveness of iCARE Nigeria. Among these strategies, six were specific to the PN role, namely selection, training, matching, and supervision of PNs, adapting communication styles to meet patient needs, and screening patients for adherence challenges. Two implementation strategies were specific to the mHealth component of the intervention: conduct and response to baseline telecommunication needs assessment and change in the distribution of funds for airtime. Our findings provided support for the use of the ERIC typology [15]. All but one of these strategies identified by our analysis (matching of PNs with peers) aligned closely with the ERIC [15]. However, the identification and sharing of novel strategies across diverse settings is also a critical process to advance the field. Future studies can additionally assess the comparative effectiveness of these strategies once elucidated.

Peer-based support strategies have been recommended in the US and international treatment guidelines to enhance ART adherence and engagement in HIV care for AYA-HIV [17,18,19]. Accordingly, peer support has increasingly been incorporated into interventions focused on improving HIV care outcomes for AYA-HIV in LMIC settings [20,21]. In our study, PNs were able to utilize their lived experience to identify adherence challenges of study participants and to work with study coordinators to address them. There is variation in descriptions of peer navigation interventions for PWH, including how they are trained, mentored, and chosen, and their frequency of interaction with peers [21,22]. Our results add to limited descriptions in the literature of how peers were matched to patients/clients—a step that might be critical for establishing rapport, leveraging commonalities, and navigating important barriers like stigma. These results highlight the importance of explicit descriptions of strategies used to implement peer navigation.

Digital health solutions, including SMS medication reminders, have enriched the toolbox of interventions available to support adherence to AYA-HIV [20,23]. mHealth strategies are appealing to youth who are often early adopters of technology, but mHealth reminders have shown mixed results for improving medication adherence and viral suppression for AYA-HIV [24,25,26,27,28,29]. Importantly, only a few of these published studies focused specifically on AYA-HIV in SSA [28]. While differences in implementation strategies for mHealth reminders and outcome measures may have contributed to heterogeneity in reported effectiveness, it is noteworthy that, unlike the other studies, TXTXT was deployed in combination with peer navigation in iCARE Nigeria, reflecting a bundled intervention delivered via a set of discrete implementation strategies.

Our analysis highlighted two implementation strategies critical for the successful implementation of iCARE Nigeria’s mHealth component and reflected the importance of understanding the telecommunication infrastructure in the study population. Conducting a baseline telecommunication needs assessment identified potential implementation barriers. Program staff were able to address challenges including offering advice for charging, and broken screens as well as providing phones at baseline. In a LMIC environment with fragile telecommunication infrastructure, this strategy may be critical for the success of mHealth interventions. Despite telecommunication infrastructure challenges within the iCARE Nigeria study, SMS reminders were sent successfully to virtually 100% of study participants, but participants replied to less than half of these reminders.

Another strategy that was described and adapted in our study was the change in airtime funds distributed to study participants. During the study, it became evident that participants had difficulty regulating their data use and depleted their airtime very early in the month. As a result, many participants could not respond to the two-way messages, jeopardizing the feasibility and fidelity of the intervention. While a description of the compensation provided to research participants is often stipulated in study methods, for mHealth interventions, documentation of both the amount and frequency of financial support for telecommunication services (i.e., airtime or data) may be important to assess the impact on implementation and indeed effectiveness outcomes [25,26,27,28]. However, it is unclear whether the low response rate to reminders also resulted from telecommunication challenges, financial barriers, habituation from the intervention, or lack of response for other reasons [30]. This is an important area for further exploration.

## 5. Conclusions

We used a mixed methods approach to identify implementation barriers and strategies employed to promote successful implementation. The approach, building on the published effectiveness results, provided insight into which core implementation strategies would likely need to be replicated or adapted to scale up the intervention across several other sites in Nigeria. One weakness of our study was that qualitative feedback from study participants could not be obtained due to constraints on in-person meetings during the COVID-19 pandemic. Further, as stigma assessments were measured at baseline, we were unable to assess the impact of the intervention on HIV-related stigma—a major barrier to treatment adherence in this population. In addition, our data was collected retrospectively which could have introduced recall bias. Nonetheless, our analysis highlights the importance of utilizing implementation research to identify and document multiple strategies used to implement evidence-based interventions and guide replication, scaling up, and adaptation of said interventions.

## Figures and Tables

**Table 1:** Description of implementation strategies responsible for effectiveness and implementation outcomes of the iCARE Nigeria treatment intervention.

Strategy	Actor	Action ^+^	Action Target	Temporality	Dose	Implementation Outcomes	Justification	Aligns with ERIC Strategy
**Facilitated Success**	Study team	Identification and **selection** of PNs from peer leaders in adolescent club	PN	Pre-implementation	Once	Feasibility and acceptability	Leverage experience and demonstrated commitment of peer club leaders to the health of their peers	YES: Identify and prepare champions
	Study team	Comprehensive **training** and program manual “bible”	PN	Pre-implementation	2-day intensive training and handbook, refresher training at week 24	Feasibility, acceptability, adoption	Address gaps in knowledge and skill for key PN functions	YES: Develop educational materials
	Study team	**Matching** PN with participants	PN	Baseline	Once	Feasibility, adoption	Ensure relatability across age-appropriate shared experiences and understanding	NO
	Program managers/study coordinator	**Supervision** of PN	PN	Implementation	Once to twice weekly	Feasibility, fidelity adoption	To provide resources to troubleshoot challenges faced by study participants, and identify/address needs of PN	YES: Provide clinical supervision
	PN	**Adapting mode of communication** to participant needs (text, voice call, WhatsApp, etc.)	Participant	Implementation	As needed	Feasibility acceptability, adoption	Ensures that communication style is feasible based on patients’ mobile phone and that communication itself is effective	YES: Promote adaptability
	PN	**Routine screening for adherence challenges**	Participant	Implementation	As needed; minimum twice monthly, maximum daily	Acceptability	Peer navigation services	YES: Intervene with patients/consumers to enhance uptake and adherence
**Addressed Barriers**	Study coordinator	**Conduct telecommunication needs assessment**	Participant	Baseline	Once	Feasibility	Identifies telecommunication barriers and allows study team to develop action plan to address	YES: Assess for readiness and identify barriers and facilitators
**Adapted to Address Barriers**	Study coordinator	**Change distribution of funds for airtime (monthly to weekly)**	Participant	Implementation	Weekly	Feasibility fidelity	Prevents patients from exhausting phone airtime at the beginning of the month and not being able to respond to the two-way SMS intervention	YES: Alter incentive/allowance structures

PN = Peer Navigator. ^+^ Core implementation strategies responsible for intervention and implementation success.

## Data Availability

The data are not publicly archived but can be provided via contacting the corresponding author.
